# Magnetoelectric effect in nanogranular FeCo-MgF films at GHz frequencies

**DOI:** 10.1016/j.jmmm.2017.08.088

**Published:** 2018-01-15

**Authors:** Kenji Ikeda, Nobukiyo Kobayashi, Ken-Ichi Arai, Shin Yabukami

**Affiliations:** a Research Institute for Electromagnetic Materials, 2-1-1 Yagiyama-minami, Taihaku-ku, Sendai 982-0807, Japan; b Tohoku Gakuin University, 1-13-1 Chuou, Tagajou-si, Miyagi 985-8537, Japan

**Keywords:** Nanogranular, Magnetoelectric effect, Dielectric relaxation, High frequency, Microstructure

## Abstract

The magnetoelectric effect is a key issue for material science and is particularly significant in the high frequency band, where it is indispensable in industrial applications. Here, we present for the first time, a study of the high frequency tunneling magneto-dielectric (TMD) effect in nanogranular FeCo-MgF films, consisting of nanometer-sized magnetic FeCo granules dispersed in an MgF insulator matrix. Dielectric relaxation and the TMD effect are confirmed at frequencies over 10 MHz. The frequency dependence of dielectric relaxation is described by the Debye-Fröhlich model, taking relaxation time dispersion into account, which reflects variations in the nature of the microstructure, such as granule size, and the inter-spacing between the granules that affect the dielectric response. The TMD effect reaches a maximum at a frequency that is equivalent to the inverse of the relaxation time. The frequency where the peak TMD effect is observed varies between 12 MHz and 220 MHz, depending on the concentration of magnetic metal in the nanogranular films. The inter-spacing of the films decreases with increasing magnetic metal concentration, in accordance with the relaxation time. These results indicate that dielectric relaxation is controlled by changing the nanostructure, using the deposition conditions. A prospective application of these nanogranular films is in tunable impedance devices for next-generation mobile communication systems, at frequencies over 1 GHz, where capacitance is controlled using the applied magnetic field.

## Highlights

• Magnetoelectric effect in the nanogranular FeCo-MgF films has been investigated.
• Magnetoelectric effect and dielectric relaxation are confirmed at frequencies over 10 MHz.
• The inter-spacing of granules and the relaxation time decrease with increasing magnetic metal concentration.
• The magnetoelectric effect reaches a maximum at a relaxation frequency.

## 1 Introduction

The magnetoelectric effect is an attractive phenomenon for material physics research, and device applications [1], [2], [3], [4]. Materials where the existence of the magnetoelectric effect has been reported include oxides and quantum dots [5], [6], [7], [8]. Since the operating temperature of these materials is much lower than room temperature, they are not suitable for use in industrial applications. We recently observed the magnetoelectric effect in nanogranular films.

The structure of the nanogranular films comprises a complex of nanometer-sized magnetic metal granules, covered with an insulating matrix. The various functional properties of the film are due to the ratio of the metal granules to the insulator matrix [9], [10], [11]. Nanogranular films with a high concentration of magnetic granules exhibit superior soft magnetism in the high frequency band, due to the strong exchange interaction between the magnetic granules, and the high resistivity of the insulator matrix [12], [13], [14], [15]. When the concentration of the insulator matrix is relatively high, the exchange interaction between neighboring magnetic granules is removed, due to the energy barrier of the insulator matrix. Thus, a charge (electron or hole) is able to transit between neighboring granules, through the thin insulator matrix, via quantum-mechanical tunneling. The electric charge tunneling process depends on the relative orientation of the magnetic moments between the granules (spin-dependent tunneling), which results in the appearance of tunneling magnetoresistance (TMR) in nanogranular films [16], [17], [18]. The tunneling current increases when the magnetic moments are aligned in parallel to the externally applied magnetic field.

Further decreases to the concentration of magnetic granules lead to a reduction of the tunneling current, due to the relatively thick insulator matrix. Recently, our group found that nanogranular films with high insulator matrix concentrations exhibit a dielectric property, and a tunneling magnetodielectric (TMD) effect, at room temperature [19]. This phenomenon is caused by electric polarization, formed by the transition of thermally-activated electrically-charged carriers between neighboring magnetic granule pairs, via quantum-mechanical tunneling [20], [21]. In this process, the transition probability of the carrier depends on the charging energy, the distance between the magnetic granules, the height of the tunneling barrier, and the relative direction of magnetization between the granule pair. The electric potential of granule pairs varies according to the direction of the applied AC electric field, due to the transition of charged carriers through the insulator barrier. The oscillation of the charging states of magnetic granule pairs results in an electric polarization. This polarization is the origin of the dielectric property in nanogranular films.

In a previous study [22], the frequency dependence of permittivity was reported only for values below 1 MHz, due to the limitations of the system used to measure capacitance. The dielectric relaxation process of nanogranular films at high frequencies is not evaluated at this stage. The existence of dielectric and TMD effects in the GHz band is important for realizing multi-functional electromagnetic devices. Measuring scattering parameters using a vector network analyzer has been established as a suitable method for determining high frequency permittivity [23].

The microstructure of nanogranular films (grain size, inter-grain spacing) is significant, since this affects electrical polarization, and electromagnetic interactions. In particular, the relaxation time, τ_r_, depends on the inter-spacing of the magnetic granules, because a reduction in tunneling barrier thickness leads to an increase in tunneling transition rate of 1/2τ_r_. During fabrication, an increase in the concentration of magnetic metal in the films results in a decrease in the inter-spacing of the granules. Conversely, a decrease in the concentration of magnetic metal leads to an increase in the inter-spacing [24]. Therefore, the relaxation time can be controlled via the inter-spacing of the granules, using the magnetic metal concentration, a deposition variable, which means that the dielectric response and the TMD effect in the high frequency band can be designed arbitrarily.

In this work, we studied the high frequency dielectric property and TMD effect in nanogranular FeCo-MgF films with various magnetic metal concentrations, by measuring scattering parameters of the films. The inter-spacing of magnetic granules and the grain size of the FeCo-MgF films were observed through high-resolution transmission electron microscopy (TEM). We report on the correlation between the high frequency dielectric property and the theory of quantum mechanical tunneling, as well as control of the TMD effect using deposition conditions.

## 2 Materials and methods

### 2.1 Thin film preparation and characterization

The nanogranular FeCo-MgF films were prepared using a tandem deposition method [25], via an RF-sputtering system with FeCo and MgF_2_ targets. The films were deposited on 50 mm × 50 mm quartz substrates in Ar atmosphere, at a pressure of 1.0 Pa. The thickness of the films was regulated to approximately 1 μm. To obtain a homogeneous granular structure, the quartz substrates were alternately rotated around the FeCo and MgF_2_ targets. The rotation speed of the substrate was kept constant at 12 RPM for each deposition. The ratio of FeCo to MgF was controlled by the power of the FeCo and MgF_2_ targets.

The composition of the nanogranular films was evaluated using wavelength dispersive X-ray spectroscopy (WDS). The microstructure of the nanogranular film was observed with high-resolution TEM. The magnetization curves were measured using a vibrating sample magnetometer (VSM). All measurements were performed at room temperature.

### 2.2 High frequency measurement of relative permittivity

**Fig. 1:**
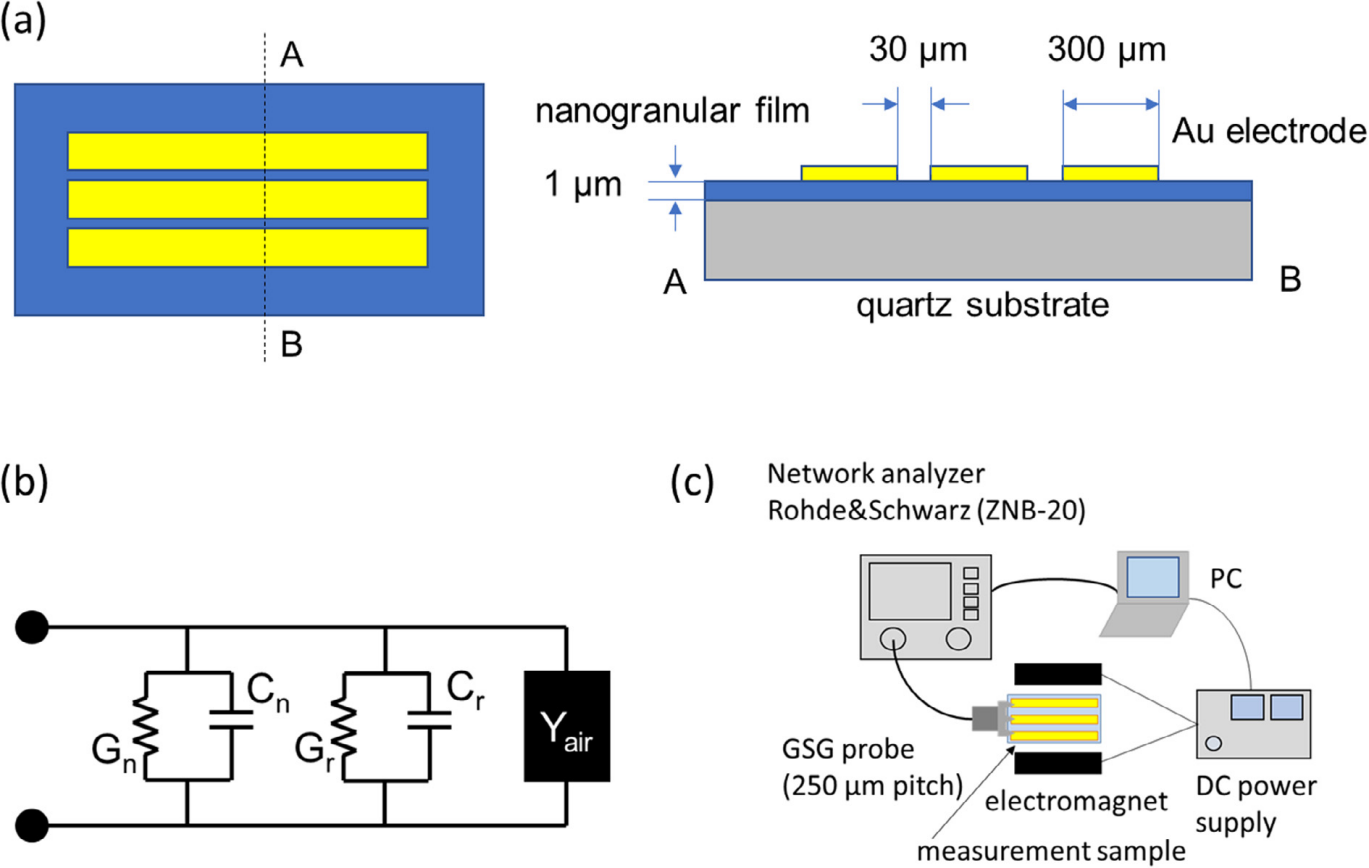
Schematic view of the high frequency measurement setup. (a) Coplanar line sample for high frequency measurement. (b) Equivalent circuit of a coplanar line with an open circuit termination. (c) Setup for measurement of the dielectric and TMD effects.

To measure the frequency dependence of the relative permittivity of the nanogranular films, a coplanar line was fabricated using the following process. An Au layer was deposited on a nanogranular film via RF-sputtering. The coplanar line was then defined using a lift-off method. A schematic view of a coplanar electrode is shown in Fig. 1a. The characteristic impedance of the coplanar line was regulated to approximately 50 Ω. Another coplanar line was fabricated directly onto a quartz substrate without a nanogranular film, for use as a reference sample. The equivalent circuit of the open circuit terminated coplanar line is shown in Fig. 1b, where G_n_ and C_n_ are the respective conductance and capacitance of the nanogranular film, G_r_ and C_r_ are the respective conductance and capacitance of the quartz substrate, and Y_air_ is a stray admittance. The capacitances (C_n_, C_r_) and conductances (G_n_, G_r_), were calculated from the reflection coefficient (S_11_) of the coplanar line, with an open circuit termination. The reflection coefficient was measured using a vector network analyzer (Rohde and Schwarz, ZNB20), and a conventional wafer probe. The frequency range was between 1 MHz and 10 GHz, with an intermediate frequency (IF) bandwidth of 10 Hz.

The relative capacitance of the coplanar lines on the nanogranular film and the reference sample, C_n_–C_r_, corresponds to the relative permittivity of the nanogranular film. The capacitance of the coplanar line on the nanogranular film was calculated using an electromagnetic field simulator. The relative permittivity of the nanogranular film was varied between 2 and 1000. The relative permittivity of the quartz substrate was fixed to 3.8.

The dependence of the permittivity of the nanogranular films on the magnetic field was evaluated with the vector network analyzer, using an electromagnet (Fig.1c). The magnetic field was varied within a range of ±80 kA/m. The change in magnetoelectric permittivity, Δε′ = ε′_80k_ − ε′_0_, was derived by subtracting the results of measurements with, and without an applied magnetic field. ε′_80k_ is the relative permittivity when the magnitude of the applied magnetic field is 80 kA/m, and ε′_0_ is the relative permittivity without a magnetic field. The TMD effect was defined as Δε′/ε′_0_.

### 2.3 Derivation of the relaxation time using a Debye-Fröhlich model

The relaxation time of the nanogranular films was derived by fitting the frequency dependence of the relative permittivity to the Debye-Fröhlich model [20], [21], [22]. When the spin-dependent tunneling process between magnetic granule pairs is applied to the Debye-Fröhlich model, the frequency dependence of the permittivity of the nanogranular films is calculated as (1):

(1) $$\varepsilon (\omega )=\varepsilon_{\infty }+\frac{\Delta \varepsilon }{1+{\left(i\omega \tau_{r}\right)}^{\beta }}\text{,}$$

where Δε is the dielectric strength, ε_∞_ is the high frequency dielectric constant, β is an exponent in the range 0 < β < 1, representing the index of the distribution of the relaxation time [26], ω is the radial frequency (2πf), and τ_r_ is the relaxation time of the spin-dependent tunneling rate.

The relaxation time depends on the magnetic direction between magnetic granule pairs, and is expressed as (2) [21], [27]:

(2) $$\frac{1}{\tau_{r}}=\frac{1}{\tau_{r0}}\left(1+P_{T}^{2}\frac{M^{2}}{{M_{s}}^{2}}\right)$$

where *P_T_* is the tunneling spin polarization, τ_r0_ is the relaxation time without magnetic field, *M* is the magnetization and *M*_s_ is the saturation magnetization. The magnitude of the TMD effect was defined and calculated using the change in the relaxation time as (3)

(3) $$\frac{\Delta \varepsilon^{\prime }(\omega \text{,}\tau )}{\varepsilon_{0}^{\prime }(\omega \text{,}\tau_{\mathrm{ro}})}=\frac{\varepsilon_{m}^{\prime }(\omega \text{,}\tau_{r})-\varepsilon_{0}^{\prime }(\omega \text{,}\tau_{r0})}{\varepsilon_{0}^{\prime }(\omega \text{,}\tau_{r0})}\text{,}$$

where *Δε*’ is the change of relative permittivity caused by magnetic field, *ε*’_m_ is the relative permittivity with magnetic field and *ε*’_0_ is the relative permittivity without magnetic field.

## 3 Results and discussion

### 3.1 Relative permittivity of nanogranular films

**Fig. 2:**
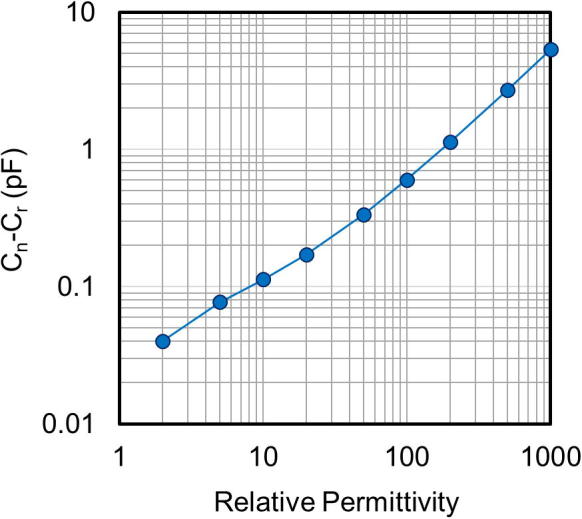
Simulated relative capacitance (C_n_–C_r_) as a function of the relative permittivity of the nanogranular films.

The relative capacitance (C_n_–C_r_) was calculated as a function of the relative permittivity, as shown in Fig. 2. The relative permittivity of the nanogranular films was derived by comparing the measured relative capacitance with this calculation [28], [29].

**Fig. 3:**
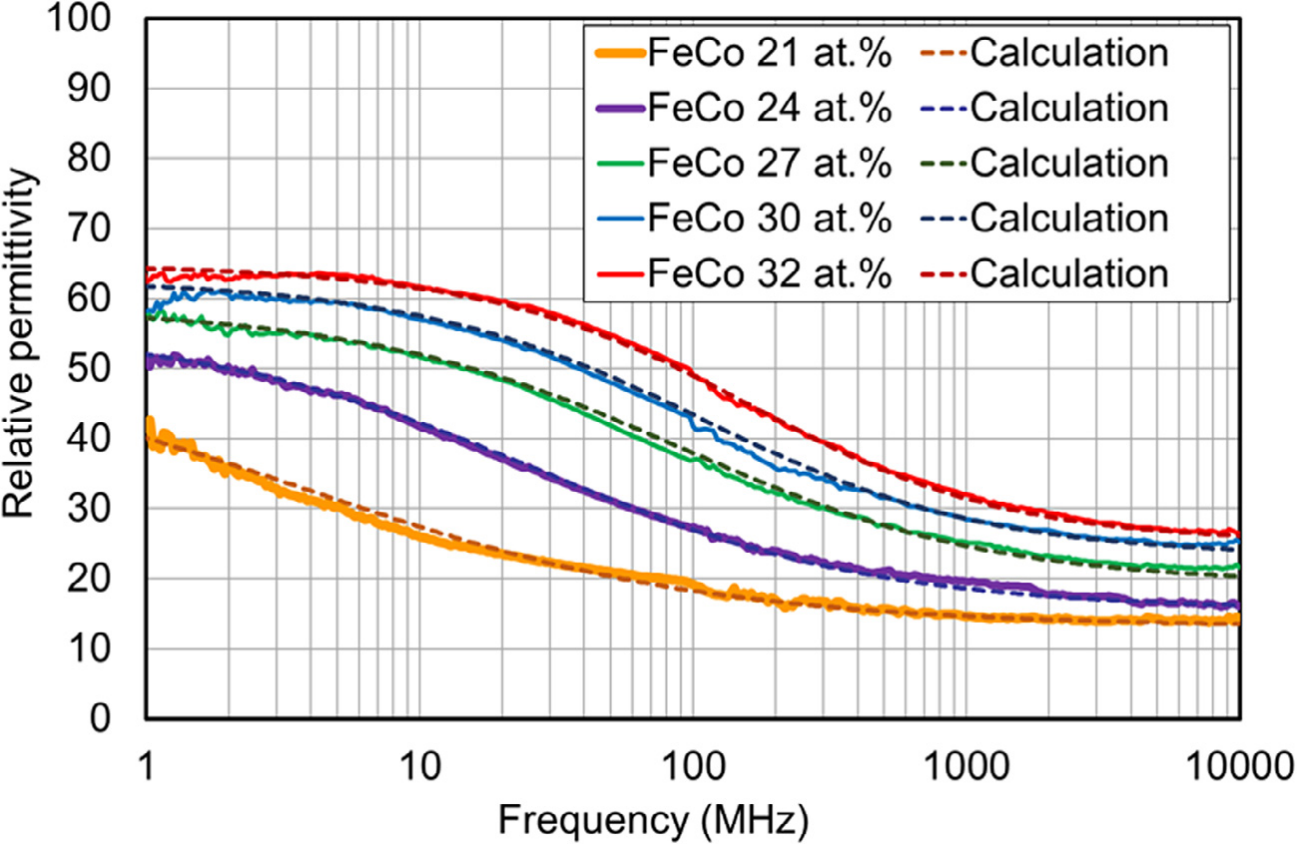
Frequency dependence of the relative permittivity, with varying FeCo content (21, 24, 27, 30, and 32 at.%).

The frequency dependence of the relative permittivity of granular films, with varying FeCo content (measured in atomic percent, at.%), appears in Fig. 3. Solid lines depict results derived by fitting the measured capacitance, C_n_–C_r_, to the simulated relative permittivity (Fig. 2), and dashed lines are results from theoretical calculations based on the spin-dependent dielectric relaxation model described by (1). The magnitude of the relative permittivity is greater than 10 at frequencies over 1 GHz, which indicates the existence of the dielectric effect in the GHz band, in nanogranular films. The relative permittivity increases with the FeCo content of the films for all frequencies considered, and decreases with increasing frequency. These results indicate that the relative permittivity in the high frequency band can be controlled using the magnetic metal concentration (FeCo content). The decrease in the relative permittivity, with increasing frequency, is thought to be caused by dielectric relaxation, characterized by a relaxation time, τ_r_. The dielectric relaxation frequency (f_r_ = 1/τ_r_) corresponding to the decrease in permittivity, migrates to the high frequency region as the FeCo content increases. We observed a good fit between results from experiments and theoretical calculations in the entire frequency range, regardless of the FeCo content, suggesting that the dielectric relaxation of the nanogranular films is broadly explained by the Debye-Fröhlich model, using the dispersion of the relaxation time.

**Fig. 4:**
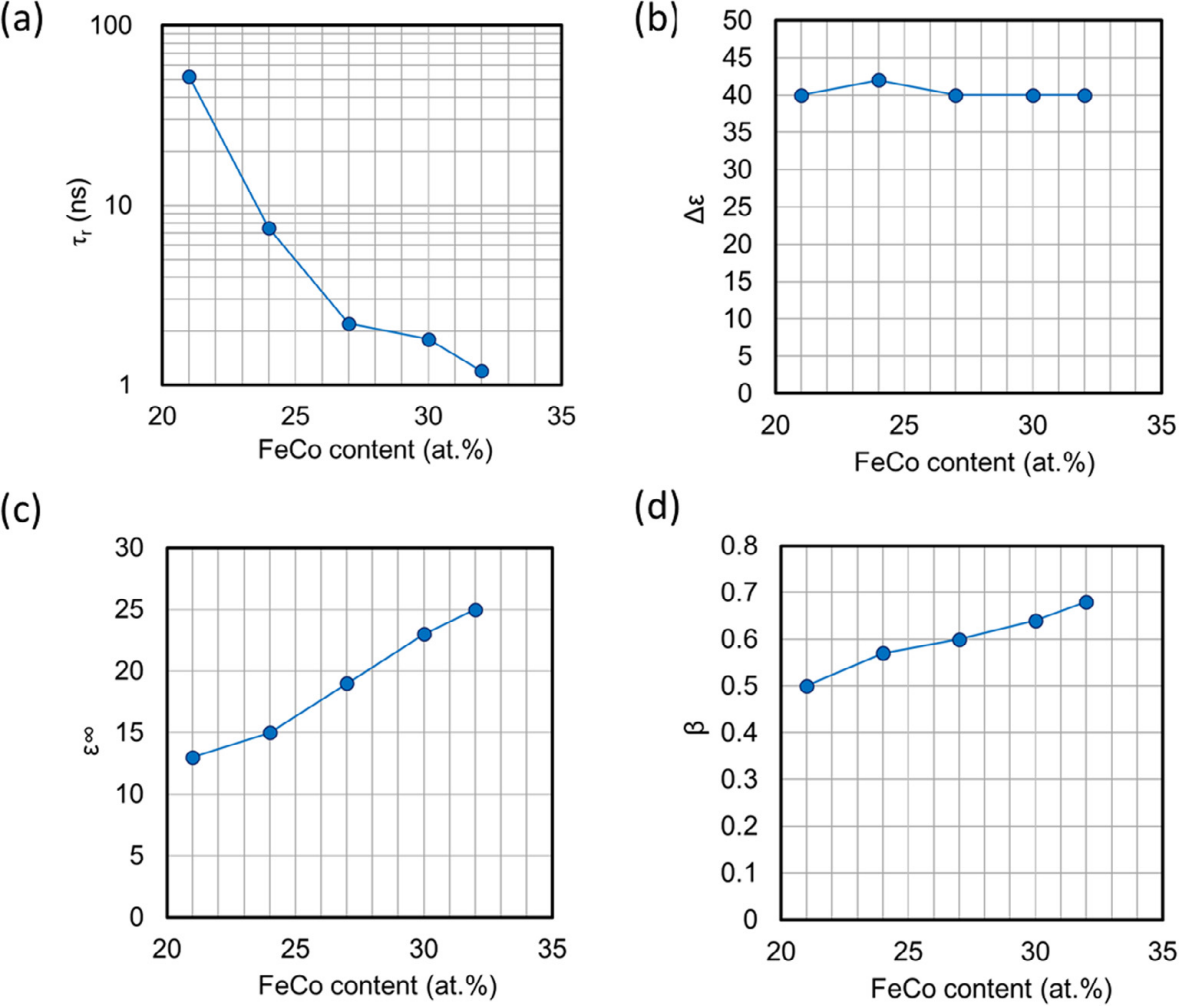
Fitting parameters, calculated using Debye-Fröhlich model, as a function of FeCo content: (a) relaxation time, τ_r_, (b) the index of the relaxation time distribution, β, (c) dielectric strength, Δε, and (d) high frequency dielectric constant, ε_∞_.

Fig. 4 shows the dependence of the fitting parameters (τ_r_, β, Δε, ε_∞_) on FeCo content, calculated using (1) (displayed as dashed lines in Fig. 3). The relaxation time decreases with increasing FeCo content (Fig. 4a). When the concentration of FeCo is over 24 at.%, the relaxation time is on the order of nanoseconds. The decrease in τ_r_ means that the dielectric relaxation frequency approaches the GHz band. The index of the distribution of the relaxation time (β) increases monotonically with FeCo content (Fig. 4b), which means that the distribution of the relaxation time narrows with increasing FeCo content. The dielectric strength (Δε) remains constant, regardless of the FeCo content (Fig. 4c), which indicates that the magnetic granule pairs constituting the electric polarization are practically invariable. The high frequency dielectric constant (ε_∞_) increases gradually with FeCo content (Fig. 4d). The relationship between the FeCo dependence of Δε and ε_∞_ can be explained by the fact that a part of Δε is transferred to ε_∞_, due to the lack of an avenue for dielectric relaxation, for frequencies over 10 GHz (upper frequency limit of this measurement).

### 3.2 Microstructure of the nanogranular films

**Fig. 5:**
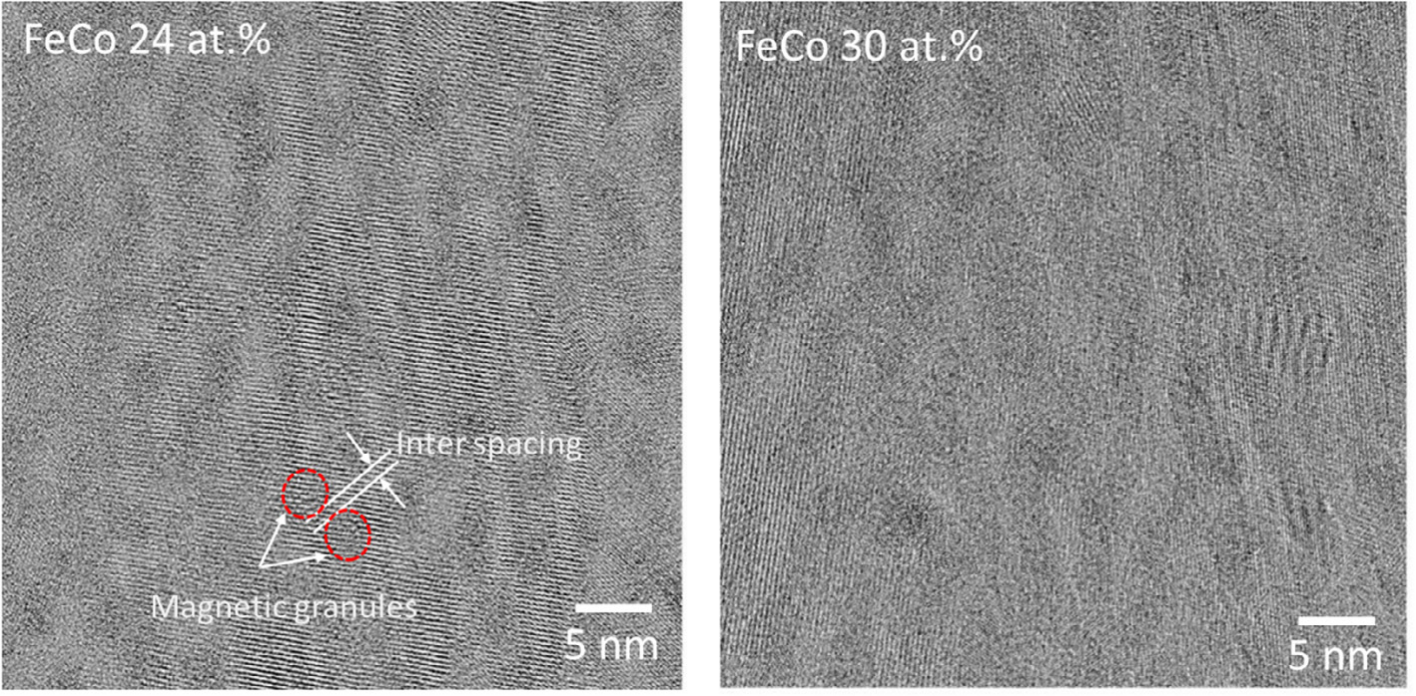
Cross-sectional TEM images of nanogranular FeCo-MgF films, with an FeCo content of 24 and 30 at.%.

High-resolution cross-sectional TEM images of nanogranular FeCo-MgF films, with an FeCo content of 24 and 30 at.%, are exhibited in Fig. 5. The dark and bright contrasts correspond to magnetic FeCo granules and the MgF matrix, respectively. These images show that nanometer-sized FeCo granules are dispersed in the MgF matrix, clearly confirming the assumed structure of the films. The size of the magnetic granules, and the distance between the granules, changes slightly with the FeCo content of the nanogranular films.

**Fig. 6:**
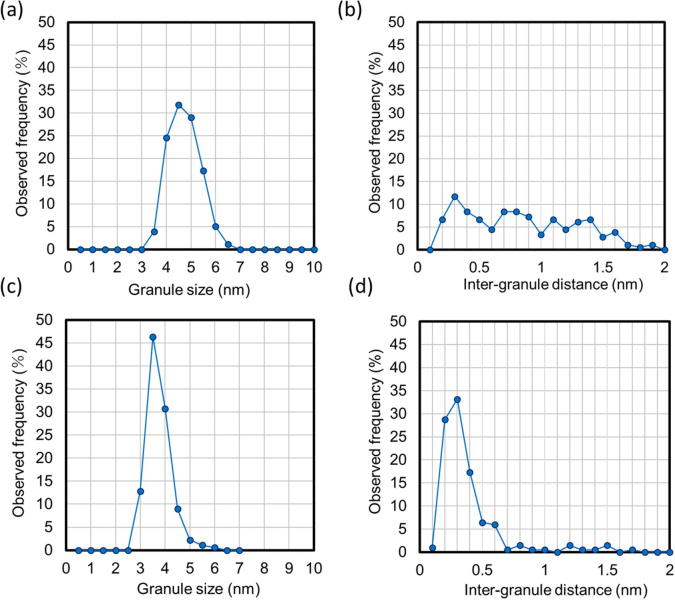
Distribution of the size of magnetic granules, and nearest neighbor distance distribution of the granules. (a) Granule size distribution (FeCo, 24 at.%), (b) nearest neighbor distance distribution (FeCo, 24 at.%), (c) granule size distribution (FeCo, 30 at.%), (d) nearest neighbor distance distribution (FeCo, 30 at.%).

Fig. 6 shows the distribution of the size of the magnetic granules, and the nearest neighbor distance distribution of the granules, estimated using TEM results (Fig. 5). The average sizes of the magnetic granules, as well as the average nearest neighbor distance of the granules are summarized, with corresponding standard deviations, in Table 1. These results indicate that increasing the magnetic metal concentration results in slighter grain growth, a notable diminution in the nearest neighbor distance of the granules, and narrowing of the dispersion of both the granule size and the nearest neighbor distance. The decrease in the nearest neighbor distance of the granules with increasing FeCo content is consistent with the response of τ_r_ (Fig. 4a), as the reduction of the tunneling barrier thickness (nearest neighbor distance) leads to the activation of a 1/2τ_r_ tunneling rate. In addition, the reduction of both the dispersion of granule size and the nearest neighbor distance implies an increase to β with FeCo content, which is in agreement with the results of calculations (Fig. 4b).

**Table 1:** Statistical summary of parameters of nanogranular films, with an FeCo content of 24 and 30 at.%, derived from analysis of TEM images.

FeCo content (at.%)	Average granule size	Average nearest neighbor distance of granules	Standard deviation of granule size	Standard deviation of nearest neighbor distance of granules
24	3.46 nm	0.80 nm	0.46	0.47
30	3.86 nm	0.33 nm	0.41	0.28

**Fig. 7:**
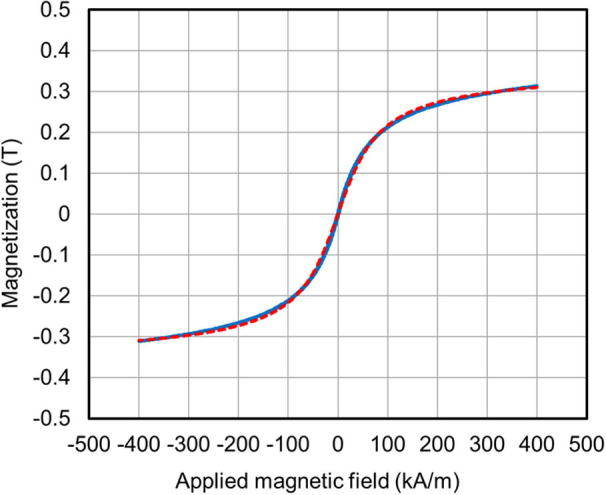
Magnetization curve of a nanogranular film (FeCo, 24 at.%).

Fig. 7 depicts the magnetization curves of a nanogranular FeCo-MgF film with 24 at.% FeCo. Results from experiments are represented by the solid blue line, and the dashed red line represents results from calculations using the Langevin function, taking the granule size distribution from the results of TEM analysis (Table 1) into account [30]. The agreement between the results of experiments and calculations, shown in Fig. 7, means that the nanogranular film is in the superparamagnetic state, and the microstructure of the film corresponds to the results of TEM observation.

### 3.3 TMD effect of the nanogranular films

**Fig. 8:**
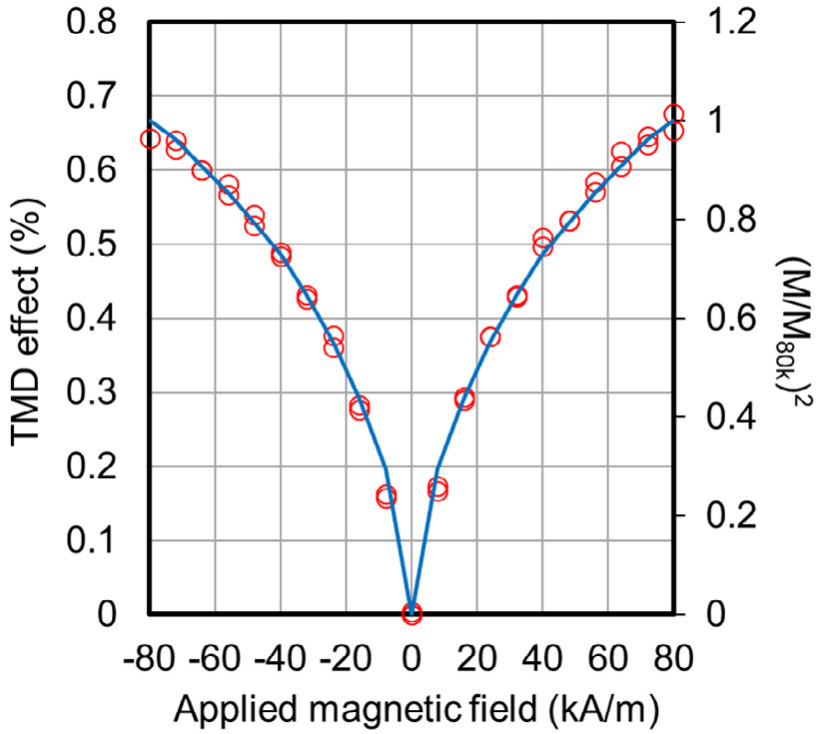
Dependence of the TMD effect of a nanogranular film (FeCo, 30 at.%), at 100 MHz, on magnetization.

The dependence of the TMD effect of a nanogranular film (FeCo content, 30 at.%), at 100 MHz, on magnetization, appears in Fig. 8. Red circles represent measurements, and blue solid lines represent (M/M_80k_)^2^ as a function of the applied magnetic field, where M_80k_ is the magnetization at 80 kA/m, the maximum applied magnetic field. The measurements coincide well with the (M/M_80k_)^2^ curve, implying that the TMD effect of the nanogranular films resulting from the applied magnetic field, can be attributed to spin-dependent carrier oscillation, caused by quantum mechanical tunneling [12], [13].

**Fig. 9:**
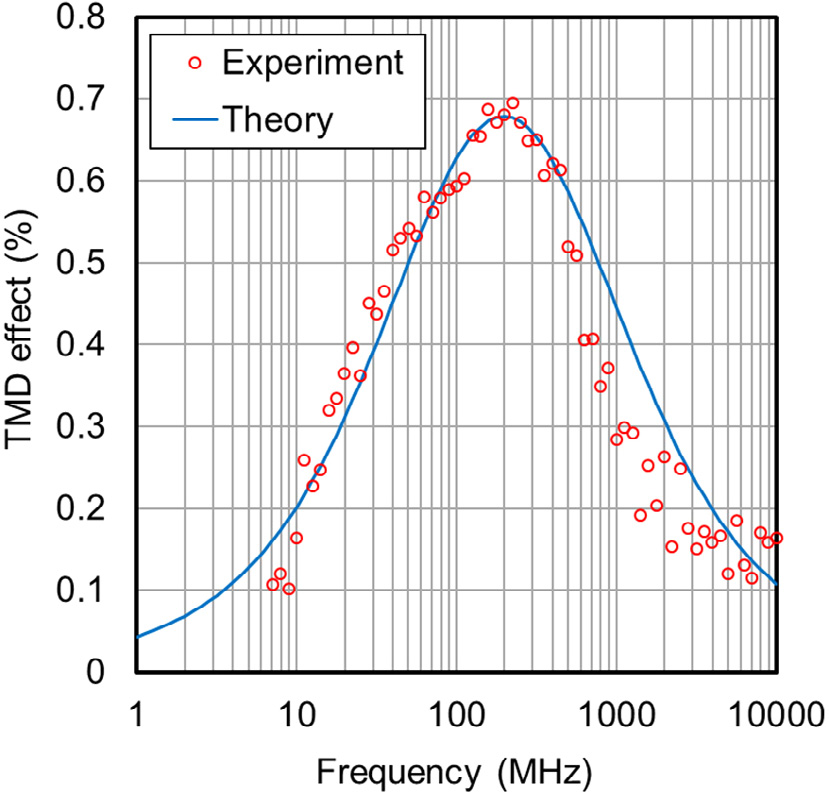
Frequency dependence of the TMD effect of a nanogranular film (FeCo, 30 at.%).

Fig. 9 shows the frequency dependence of the TMD effect of a nanogranular film with an FeCo content of 30 at.%. Red circles correspond to measurements, and the solid blue line represents theoretical calculations, described in (3), made using the magnetization dependence of the relaxation time, described in (2), and the frequency dependence of permittivity, described in (1). The maximum magnitude of the TMD effect of this film is approximately 0.7%, at 220 MHz. In the GHz band, the TMD effect varies between 0.1% and 0.3%. The accord between the measured value and the calculation, confirms the accuracy of the relaxation time derived by fitting the frequency dependent relative permittivity to the Debye-Fröhlich model (Fig. 4a), as the frequency corresponding to the peak TMD effect increases with the relaxation frequency (1/*τ*_r_).

The maximum values of the TMD effect, and corresponding peak frequencies, are listed in Table 2. The peak frequency of the TMD effect increases with the FeCo content of the films, which implies that this phenomenon can be controlled by managing the magnetic metal concentration. There is an inconsistency in the maximum values of TMD observed, which may be attributed to the existence of a ferromagnetic component due to the wide dispersion of magnetic granules. On the other hand, the frequency corresponding to the peak TMD effect is consistent with the FeCo dependence of the relaxation frequency (1/*τ*_r_: Fig. 4a), as the frequency corresponding to the peak TMD effect increases with the relaxation frequency (1/*τ*_r_). These results support the measurement method and frequency dependent permittivity, and the theoretical model used in this study. Further control of the microstructure, especially the inter-spacing of the magnetic granules, could expand the frequency band where the dielectric response and TMD effect is present. TMD effect in the nanogranular films is enhanced by an increase in relaxation time change, which implies large TMD effect will be realized by applying high tunneling spin polarization (*P_T_*) materials such as heusler alloy.

**Table 2:** Maximum value of TMD effect, and corresponding peak frequency.

FeCo content (at.%)	Peak frequency (measured)	Peak frequency (calculation)	Maximum Δε (measured)	Maximum Δε (calculation)
21	11.2 MHz	12.6 MHz	0.56%	0.61%
24	63.1 MHz	70.8 MHz	0.48%	0.58%
27	141 MHz	178 MHz	0.56%	0.56%
30	220 MHz	200 MHz	0.70%	0.67%
32	251 MHz	282 MHz	0.72%	0.68%

## 4 Conclusion

The frequency dependence of permittivity, microstructure (granule size, inter-grain spacing), and TMD effect of nanogranular FeCo-MgF films, were studied in this paper. Dielectric relaxation in the high frequency band was measured using a scattering parameter method, and explained using the Debye-Fröhlich model, with a relaxation time, τ_r_. Observation of the microstructure revealed the decrease in inter-granule spacing with increasing FeCo content, which corresponds to the trend suggested by the relaxation time derived from the high frequency permittivity measurement. The frequency corresponding to the peak TMD effect as a function of FeCo content is in accordance with the change of relaxation frequency (1/τ_r_), which is evidence that the permittivity of the nanogranular films can be attributed to quantum-mechanical tunneling between magnetic granule pairs. The frequency dependence of the relative permittivity, the dielectric relaxation, and the TMD effect all strongly depend on the FeCo content of the nanogranular films, which also exhibits a convertible magnetoelectric effect caused by the control of the magnetic metal concentration. Nanogranular films with the TMD effect are useful for high-frequency applications, such as tunable impedance devices for next-generation mobile communication systems.
